# Real-world outcomes of patients with advanced intrahepatic cholangiocarcinoma treated with programmed cell death protein-1-targeted immunotherapy

**DOI:** 10.1080/07853890.2022.2048416

**Published:** 2022-03-11

**Authors:** Min Deng, Shaohua Li, Qiaoxuan Wang, Rongce Zhao, Jingwen Zou, Wenping Lin, Jie Mei, Wei Wei, Rongping Guo

**Affiliations:** a Department of Liver Surgery, Sun Yat-sen University Cancer Center, Guangzhou, China; b State Key Laboratory of Oncology in South China, Collaborative Innovation Center for Cancer Medicine, Guangzhou, China; c Department of Radiation Oncology, Sun Yat-sen University Cancer Center, Guangzhou, China

**Keywords:** Intrahepatic cholangiocarcinoma, immunotherapy, PD-1, real-world, outcome

## Abstract

**Objective:**

There is a lack of effective treatment to improve the prognosis of intrahepatic cholangiocarcinoma (ICC). Programmed cell death protein-1 (PD-1)-targeted immunotherapy has shown promising results in a variety of malignant tumours. However, in patients with advanced ICC, the safety and efficacy of anti-PD-1 agents remain unclear.

**Methods:**

Forty-two advanced ICC patients treated with anti-PD-1 agents from August 2018 to December 2020 were retrospectively analyzed. Tumour response, overall survival (OS), progression-free survival (PFS), and time to tumour progression (TTP) were evaluated. Adverse events were also recorded.

**Results:**

The median duration of follow-up was 12.1 months, and the median time of treatment was 6.7 months for all patients. The median OS, median PFS, and median TTP for the whole cohort were 19.3 months, 11.6 months, and 11.6 months, respectively. The overall response rate (ORR) and disease control rate (DCR) for the whole cohort were 23.8% and 85.7%, respectively. Of the 42 evaluable individuals, two (4.8%) had hyperprogressive disease. The most common adverse events (AEs) were pain (*n* = 6; 14.3%), anorexia (*n* = 4; 9.5%), hypertension (*n* = 4; 9.5%), pyrexia (*n* = 3; 7.1%), cough (*n* = 3; 7.1%), and hypothyroidism (*n* = 3; 7.1%). The median OS of patients with albumin-bilirubin (ALBI) grade 1 was longer than that of patients with ALBI grade 2 (19.3 months vs. 14.7 months). The median PFS did not show a significant difference between ALBI grade 1 and grade 2 patients (13.6 months vs. 6.9 months).

**Conclusions:**

PD‐1‐targeted immunotherapy showed promising efficacy and safety in advanced ICC patients.

**Key messages**
- PD-1-targeted immunotherapy is a safe and effective treatment for advanced ICC patients.
- This study provides therapeutic strategy for advanced ICC patients.

## Introduction

Intrahepatic cholangiocarcinoma (ICC), one of the primary liver cancers, is the second most common liver malignancy after hepatocellular carcinoma (HCC) [1,2]. Owing to early local invasion, distant metastasis, and lack of effective treatment, the prognosis of ICC is still poor [3,4]. At present, only a small number of patients with ICC have the opportunity to undergo curative resection. However, even with radical resection, it is challenging for patients achieve a median survival of more than 30 months [3]. Especially for patients with advanced ICC, few effective therapies can improve the clinical prognosis [5]. Consequently, more efficient treatments are urgently needed for ICC patients.

In recent years, immune checkpoint inhibitors (ICIs), specifically, programmed cell death protein-1 (PD-1)/programmed cell death protein ligand-1 (PD-L1) inhibitors, have achieved satisfactory results in treating some malignancies [6–8].

Research has shown that high PD-L1 expression can be detected in occupational cholangiocarcinoma (cholangiocarcinoma caused by occupational exposure to organic solvents), suggesting that the critical mechanism for forming occupational cholangiocarcinoma may be related to the immune escape of the PD-1/PD-L1 pathway [9]. Another ICC study reported that the upregulation of PD-1/PD-L1 expression in tumour tissues was relevant to tumour differentiation and American Joint Committee on Cancer (AJCC) staging [10]. In addition, a survey of 54 ICC patients showed that high expression of PD-L1 in tumours was associated with lower overall survival (OS) [11].

In current clinical practice, although ICIs have been widely used in HCC, PD-1/PD-L1 inhibitors are still rarely applied to ICC, and their efficacies in the real world are unknown. Noticeably, immunotherapy seems to have therapeutic prospects in biliary tract cancer, but more studies are needed to confirm it [12–16]. Therefore, we designed this study to evaluate the safety and efficacy of anti-PD-1 immune therapy in a real-world treatment cohort of advanced ICC patients in our cancer centre.

## Patients and methods

### Study design and patients

Our study is a single-centre retrospective analysis of advanced ICC patients who received anti-PD-1 agents at Sun Yat-sen University Cancer Centre (Guangzhou, China). Patients with radiologically or histologically confirmed advanced ICC who received PD-1 inhibitor monotherapy with nivolumab, pembrolizumab, toripalimab, camrelizumab, sintilimab, or tislelizumab or a combination therapy with other modes of cancer treatments were eligible. Histopathological diagnosis of ICC was based on the World Health Organization criteria [17]. The patient’s informed consent and confidentiality of information were obtained before the treatment. The retrospective analysis was approved by the Clinical Research Ethics Committee of Sun Yat-sen University Cancer Centre (No. B20203180). The Declaration of Helsinki on biomedical research involving human participants has also been followed.

### Dosing of PD-1 inhibitor therapy

Nivolumab and toripalimab were intravenously administered at a fixed dose of 240 mg every 3 weeks. Pembrolizumab, camrelizumab, sintilimab, and tislilizumab were given at a fixed dose of 200 mg every 3 weeks intravenously.

### Assessments

Patient information was collected before and after PD-1 inhibitor treatment. Clinicopathological data are shown in Table 1. In addition, tumours were evaluated by abdominal computed tomography (CT)/magnetic resonance imaging (MRI) at baseline. Tumour size, the number of nodules, vascular invasion, and tumour metastasis were assessed based on CT/MRI. Patients underwent CT or MRI examinations 4–8 weeks after the initiation of therapy and approximately every 2 months after evaluating the treatment efficacy. Hyperprogression was defined as a progressive disease in the first radiological assessment during immunotherapy (RECIST version 1.1 [18]) with a delta tumour growth rate of >50%, corresponding to an absolute increase in the tumour growth rate exceeding 50% per month.

**Table 1.** Baseline characteristics.

	*N* = 42
Age (y), mean ± SD	55.5 ± 10.5
Gender	
Male	28 (66.7%)
Female	14 (33.3%)
Aetiology	
Hepatitis B	19 (45.2%)
Non-Hepatitis B	23 (54.8%)
Child‐Pugh stage	
A	42 (100%)
5	38 (90.5%)
6	4 (9.5%)
ECOG PS	
0	5 (11.9%)
1	35 (83.3%)
2	2 (4.8%)
BCLC stage	
A	1 (2.4%)
B	4 (9.5%)
C	37 (88.1%)
Extrahepatic metastasis	32 (76.2%)
Lymph node metastasis	18 (42.9%)
Organ metastasis	6 (14.3%)
Lymph node metastasis and organ metastasis	8 (19.0%)
Number of tumour	
Single	12 (28.6%)
Multiple	30 (71.4%)
Tumour size (cm)	7.6 ± 3.2
AFP	
<400 (ng/ml)	32 (76.2%)
≥400 (ng/ml)	10 (23.8%)
PIVKA-II	
≤40 (mAU/mL)	24 (57.1%)
>40 (mAU/mL)	18 (42.9%)
CA19-9	
≤35 (U/ml)	19 (45.2%)
>35 (U/ml)	23 (54.8%)
Immunotherapy as systemic	
First‐line	35 (83.3%)
Second‐line	7 (16.7%)
Prior treatment	
Surgery	7 (16.7%)
TACE	10 (23.8%)
HAIC	22 (52.4%)
Radiation	2 (4.8%)
Chemotherapy	4 (9.5%)
Previous target therapy	7 (16.7%)
Other treatments after PD-1 or combination	
Surgery	8 (19.0%)
TACE	10 (23.8%)
HAIC	20 (47.6%)
Ablation	1 (2.4%)
Radiation	4 (9.5%)
Chemotherapy	3 (7.1%)
Sorafenib	1 (2.4%)
Lenvatinib	20 (47.6%)
Apatinib	15 (35.7%)
Regorafenib	1 (2.4%)

ECOG PS: Eastern Cooperative Oncology Group Performance Status; BCLC: Barcelona‐Clinic Liver Cancer; AFP: alpha‐fetoprotein; PIVKA-II: protein induced by vitamin K absence or antagonist-II; CA19-9: Carbohydrate antigen 19-9; TACE: transcatheter arterial chemoembolization; HAIC: hepatic artery infusion chemotherapy.

The modified Response Evaluation Criteria in Solid Tumours (mRECIST [19]) was used to evaluate tumour response, including the following types: (I) complete response (CR); (II) partial response (PR); (III) stable disease (SD); and (IV) progressive disease (PD).

The endpoints, including overall survival (OS), progression-free survival (PFS), time to progression (TTP), objective response rate (ORR), and disease control rate (DCR), were examined in this study. OS was the time from the beginning of immunotherapy to death. Individuals who were still alive were censored at the date of the last follow-up or data cut‐off. PFS was the time from first receiving PD-1 inhibitor to disease progression, confirmed by radiography, or death, whatever came first. Subjects who were still alive and without radiologically confirmed progression were censored at the date of last contact or data cut‐off. TTP was the time from the date of first immunotherapy to initial confirmed tumour progression by imaging tests. Data from participants who died without radiologically confirmed tumour progression were censored at the last radiological evaluation date.

Albumin-bilirubin (ALBI) grade is a useful indicator for objective evaluation of liver function [20]. Studies have revealed that the ALBI grade can more accurately predict the patient’s prognosis and OS than the Child-Pugh grade [21,22]. The ALBI grade can be divided into 3 levels: grade 1, grade 2, and grade 3. The higher the score, the worse is the liver function [20,23]. The ALBI score was calculated from the formula ALBI score=(log10 bilirubin*0.66)+(albumin*−0.085), where grade 1 is ≤ −2.60, grade 2 is −2.60 to −1.39, and grade 3 is > −1.39 [20].

### Statistics

Descriptive statistics were used to analyze baseline characteristics, radiological tumour response, and adverse events. The comparison of nominal data used the chi-square test or Fisher’s exact test. Survival curves were calculated using the Kaplan–Meier method and compared utilizing the log-rank test. *p* < .05 was considered to be statistically significant. Comparisons among the six PD-1 drug groups were not conducted because the sample size was smaller after the cohort was subdivided. Statistical analyses were performed using IBM SPSS Statistics (version 26.0, SPSS Inc., Chicago, IL).

## Results

### Patients

This research included 42 ICC patients who received PD-1-targeted immunotherapy between August 2018 and December 2020. The data collection cut-off date was 31 December 31 2020. All 42 patients had at least one follow‐up imaging assessment after receiving PD-1 immunotherapy to evaluate tumour response. Twenty-eight male and 14 female ICC patients with a median age of 55.5 years old (range from 29 to 75 years old) were enrolled.

At the beginning of PD-1 treatment, 42 ICC patients used different PD-1 antibodies as follows: nivolumab (*n* = 3), pembrolizumab (*n* = 11), sintilimab (*n* = 14), toripalimab (*n* = 9), camrelizumab (*n* = 4), and tislelizumab (*n* = 1). Immunotherapy was discontinued mainly due to radiological or clinical disease progression and adverse events. Immunotherapy was used as systemic first‐line and second‐line treatment in 35 (83.3%) and 7 (16.7%) patients, respectively. The patients’ main baseline features, including age, sex, aetiology, Child-Pugh stage, ECOG PS, BCLC stage, tumour characteristics, serum tumour marker level, and previous and subsequent treatments, are shown in Table 1. The median duration of follow-up was 12.1 (95%CI: 9.9–14.3) months for all patients. The median number of cycles of PD-1 therapy at the cut-off was 6.5 (95%CI: 6.2–9.9) for all patients. The median time of treatment was 6.7 (95%CI, 5.8‐9.7) months for all patients. At the data cut-off, 9 (21.4%) patients were still receiving treatment with PD-1 immunotherapy.

### Efficacy

Of the 42 patients, four (9.5%) participants achieved complete response (CR), and six (14.3%) patients achieved partial response (PR), resulting in an overall response rate (ORR) of 23.8%. Twenty-six (61.9%) patients showed stable disease (SD), and six (14.3%) patients had progressive disease (PD) at radiological evaluation. The disease control rate (DCR) was 85.7%. The results are shown in Table 2. None of the patients were evaluated as having hyperprogression in this study.

**Table 2.** Tumour responses and survival.

	All patients (n = 42)	Nivolumab	Pembrolizumab	Toripalimab	Camrelizumab	Sintilimab	Tislelizumab
Tumour response							
CR	4 (9.5%)	0	0	1 (11.1%)	0	3 (21.4%)	0
PR	6 (14.3%)	0	1 (9.1%)	2 (22.2%)	2 (50%)	1 (7.1%)	0
SD	26 (61.9%)	3 (100%)	9 (81.8%)	6 (66.7%)	1 (25%)	7 (50%)	0
PD	6 (14.3%)	0	1 (9.1%)	0	1 (25%)	3 (21.4%)	1 (100%)
ORR (CR + PR)	10 (23.8%)	0	1 (9.1%)	3 (33.3%)	2 (50%)	4 (28.6%)	0
DCR (CR + PR + SD)	36 (85.7%)	3 (100%)	10 (90.9)	9 (100%)	3 (75%)	11 (78.6%)	0
PFS, median (95%CI)	11.6 (95%CI, 7.9–15.3)						
TTP, median (95%CI)	11.6 (95%CI, 7.9–15.3)						
OS, median (95%CI)	19.3 (95%CI, 14.9–23.7)						
6 months survival rate	90.5%						
1‐year survival rate	77%						
18 months survival rate	57%						
2‐year survival rate	19%						

CR: complete response; PR: partial response; SD: stable disease; PD: progressive disease; ORR: overall response rate; DCR: disease control rate; PFS: progression‐free survival; TTP: time to progression; OS: overall survival.

**Figure 1.**
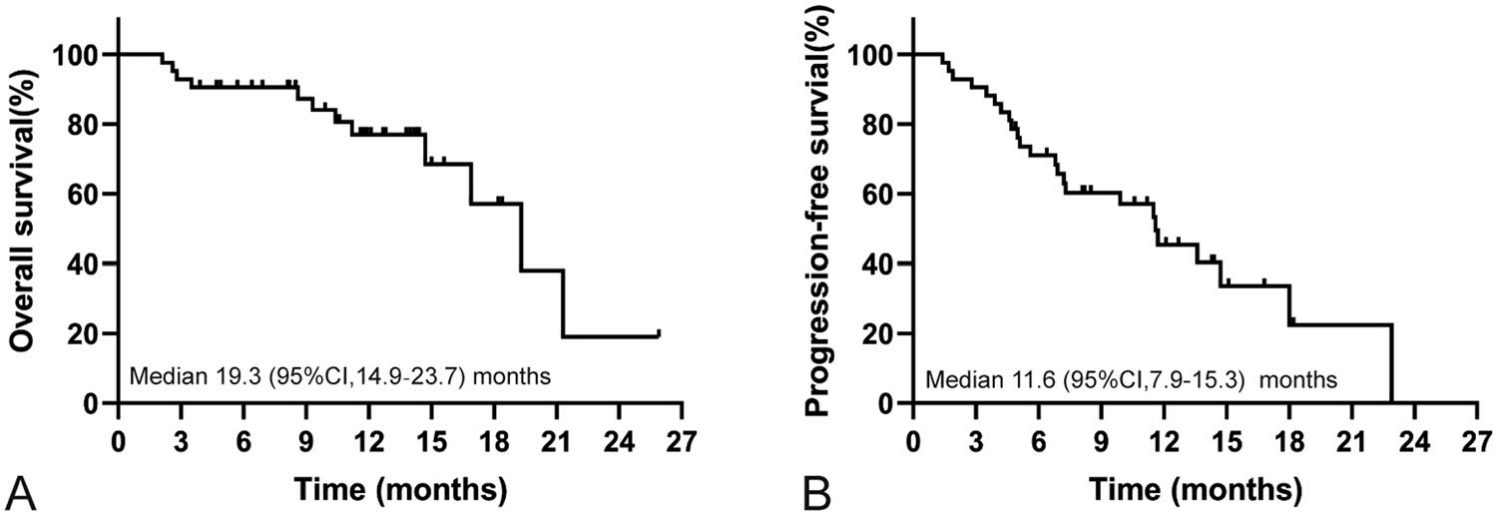
Kaplan–Meier curve showing OS and PFS for the whole cohort of patients treated with programmed cell death protein-1 (PD-1)-targeted immunotherapy. (A) OS rates in patients with ICC receiving anti-PD-1 agents. (B) PFS rates in patients with ICC receiving anti-PD-1 agents.

**Figure 2.**
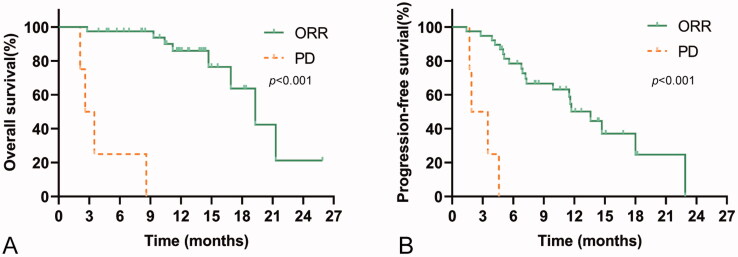
Kaplan–Meier curves showing OS and PFS for patients treated with PD-1-targeted immunotherapy according to radiological tumour response (partial response (PR)/stable disease (SD) vs. progressive disease (PD)). (A) OS rates in patients with or without disease progression. (B) PFS rates in patients with or without disease progression.

Overall, 19 (45.2%) patients had radiological disease progression when receiving PD-1-targeted immunotherapy, and 12 (28.6%) participants died during follow‐up. The median TTP was 11.6 (95%CI, 7.9–15.3) months for the whole cohort. The median OS was 19.3 (95%CI, 14.9–23.7) months for the whole cohort (Figure 1(A)). PFS was 11.6 (95%CI, 7.9–15.3) months for the entire group (Figure 1(B)), and TTP was equal to PFS. The median OS and median PFS for patients with non-PD were 19.3 (95%CI, 14.9–23.8) months and 13.6 (95%CI, 10.8–16.4) months, and the median OS and median PFS for patients with PD were 3.1 (95%CI, 1.2–4.0) months and 1.9 (95%CI, 0.1–3.7) months, respectively. The OS and PFS in patients with non-PD were significantly longer than those in patients with PD (Figure 2, All *p* < .001).

**Figure 3.**
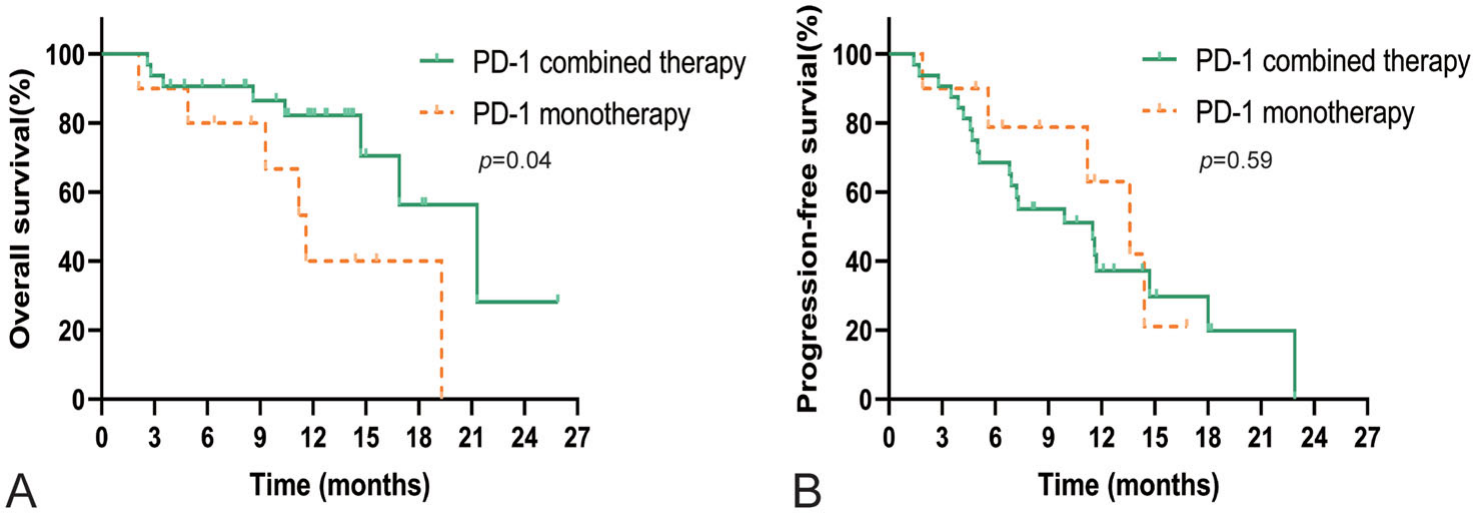
Kaplan–Meier curves showing OS and PFS for ICC patients treated with PD-1 monotherapy and PD-1 combination therapy. (A) OS rates in patients with PD-1 monotherapy and PD-1 combined with other treatments. (B) PFS rates in patients with PD-1 monotherapy and PD-1 combination therapy.

The median OS of PD-1 monotherapy (11.6 months, 95%CI, 8.6–14.6) was significantly shorter than that of PD-1 combined with other treatments (21.3 months, 95%CI, 14.7–27.9), such as transcatheter arterial chemoembolization (TACE) and hepatic artery infusion chemotherapy (HAIC) (*p* = .04). The median PFS was 10.6 (95%CI, 8.5–13.5) months and 11.5 (95%CI, 5.5–17.5) months for PD-1 monotherapy and PD-1 combined therapy, respectively (*p* = .59). The results are shown in Figure 3.

### Safety

Twenty‐one (50%) patients experienced at least one adverse event (AE). Grade ≥ 3 events occurred in two patients (4.8%), and grade 1 or grade 2 events occurred in 21 patients (50%). The most common adverse events were pain (*n* = 6; 14.3%), anorexia (*n* = 4; 9.5%), hypertension (*n* = 4; 9.5%), pyrexia (*n* = 3; 7.1%), cough (*n* = 3; 7.1%), and hypothyroidism (*n* = 3; 7.1%). Two (4.8%) patients developed adverse events of higher grade (grade ≥ 3), including pain (*n* = 1; 2.4%) and hepatitis (*n* = 1; 2.4%). PD-1 immunotherapy was discontinued, and corticosteroids were used in one patient with hepatitis. The remaining adverse events were treated symptomatically (*n* = 19; 45.2%). All adverse events observed in PD-1-treated patients are shown in Table 3. No patient died due to PD-1 immunotherapy during treatment. A dose delay due to adverse events was required in eight (19%) participants receiving PD-1 immunotherapy; of these patients, three (7.1%) were treated with pembrolizumab, three (7.1%) with sintilimab, one (2.4%) with nivolumab, and one (2.4%) with camrelizumab.

**Table 3.** Adverse events.

Effect	All patients (*n* = 42)	
	Any grade	Grad*e* ≥ 3
Pyrexia	3 (7.1%)	–
Rash	1 (2.4%)	–
Fatigue	1 (2.4%)	–
Anorexia	4 (9.5%)	
Palpitation	1 (2.4%)	–
Hiccups	1 (2.4%)	–
Nausea	2 (4.8%)	–
Vomiting	2 (4.8%)	–
Pain	6 (14.3%)	1 (2.4%)
Headache	1 (2.4%)	
Low back pain	1 (2.4%)	–
Cough	3 (7.1%)	–
Diarrhoea	2 (4.8%)	–
Abdominal distension	1 (2.4%)	
Arthralgia	1 (2.4%)	–
Dental ulcer	2 (4.8%)	–
Hypothyroidism	3 (7.1%)	–
Hypertension	4 (9.5%)	
Constipation	2 (4.8%)	–
Hypoproteinemia	1 (2.4%)	–
Myelosuppression	2 (4.8%)	–
Platelet count decrease	2 (4.8%)	
Immune associated pneumonia	1 (2.4%)	–
Hand-foot skin reaction	1 (2.4%)	–
Hepatitis	2 (4.8%)	1 (2.4%)
Peripheral neuropathy	1 (2.4%)	–

### Efficacy and safety according to ALBI grade

**Figure 4.**
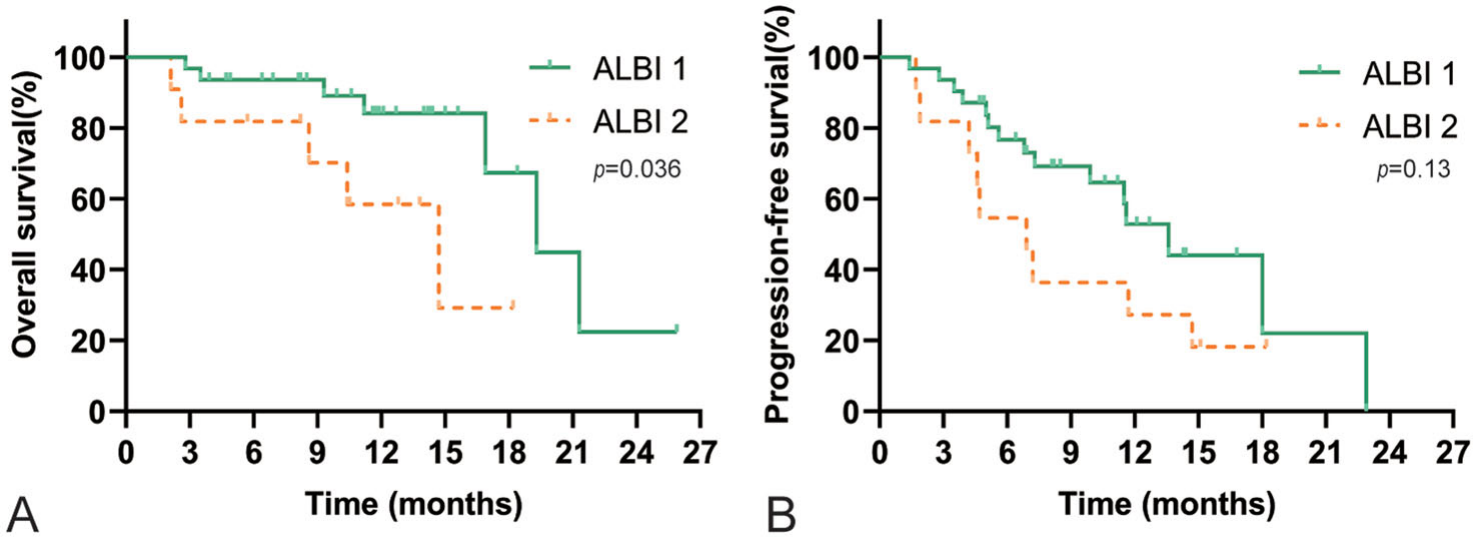
Kaplan–Meier curves showing OS and PFS for patients with different ALBI grades. (A) OS rates in patients with ALBI grade 1 and grade 2. (B) PFS rates in patients with ALBI grade 1 and grade 2.

The efficacy and safety of immunotherapy in patients with ALBI grades 1 and 2 were assessed. The ORR and DCR for ALBI grade 1 vs grade 2 were 19% vs 4.8% and 66.7% vs 19.0%, respectively. The median OS in patients with ALBI grade 1 (19.3 months, 95%CI, 14.8–23.8) was significantly longer than that of patients with ALBI grade 2 (14.7 months, 95%CI, 8.3–21.1) (*p =* .036). However, the median PFS in patients with ALBI grade 1 (13.6 months, 95%CI, 10.2–17.0) was not significantly different from that in patients with ALBI grade 2 (6.9 months, 95%CI, 4.1–9.7) (*p =* .13). The results are shown in Figure 4.

## Discussion

Our single-centre retrospective study proved that PD-1-targeted immunotherapy exhibited promising efficacy and slight toxicity in a real-world cohort of patients with advanced ICC. The median OS and PFS were 19.3 months and 11.6 months, respectively, for the whole cohort. The median OS and median PFS of the patients with ORR in this research were significantly longer than those of the PD group (19.3 months vs. 3.1 months, 13.6 months vs. 1.9 months). There are few studies on PD-1 in the treatment of ICC patients in China. Moreover, the background of ICC disease in China is different from that in other countries. ICC patients in China often have bile duct stones, cholangitis, or HBV infection, while ICC patients in western countries, South Korea and Japan often have primary sclerosing cholangitis as the disease background [2,24–26]. The therapeutic efficacy of PD-1 in ICC patients in different countries may be diverse, and there is still no definite evidence. Therefore, our study may provide some evidence for the treatment of PD-1 in ICC with bile duct stones, cholangitis, and HBV background. In addition, the predictive effect of ALBI grade on PD-1 treatment in ICC patients was also analyzed. This study also evaluated the different efficacy between PD-1 combined with other therapies and PD-1 monotherapy in the treatment of ICC. ALBI grade is an index that can objectively evaluate liver function and accurately predict the prognosis of patients with liver disease. ALBI grade plays a potential role in predicting prognosis in patients with advanced HCC receiving immunotherapy [27,28]. At present, there are no effective clinical indicators that can predict the efficacy of immunotherapy in ICC patients. ICC and HCC are both types of primary liver cancer. Tumour development and antitumor treatments may affect liver function. Accordingly, this study aims to use ALBI grade to predict the efficacy of immunotherapy and the prognosis of ICC patients. For the comparison between patients with different ALBI scores, the median OS of patients with ALBI grade 1 was longer than that of patients with ALBI grade 2 (19.3 months vs. 14.7 months). The median PFS did not show a significant difference between ALBI grade 1 and grade 2 patients, but the PFS of patients with ALBI grade 1 tended to be longer than that of patients with ALBI grade 2 (13.6 months vs. 6.9 months). Most of the adverse events relevant to PD-1 in this study were manageable, and only two patients developed severe adverse events.

Current studies show that ICIs combined with other medications may improve the therapeutic efficacy towards malignant tumours [29–31]. Encouragingly, ICI monotherapy or combination therapy has shown particular curative efficacy in the most common liver cancer, HCC [32–34]. In this study, the median OS of patients with PD-1 combined therapy was longer than that of patients with PD-1 monotherapy (21.3 months vs. 11.6 months). Regretfully, the median PFS between the two groups was not significantly different (11.5 months vs. 10.6 months), which may be due to the small sample size. However, from the perspective of the trend, the combined treatment has a better efficacy. Owing to the small sample size of patients with ICC, our study did not compare the efficacy of various combination therapies or analyze when the combined treatment with a PD-1 agent is the best. Current studies have shown that the efficacy of combining other therapies at the beginning of PD-1 treatment is better. The original treatment plan should be replaced when the disease progresses after the combined treatment. From the above studies, it can be seen that ICIs have achieved breakthrough results in the treatment of advanced HCC; nevertheless, the ORR and OS of single-agent therapy need to be improved. Combination therapy may be the inevitable direction of ICIs in the treatment of advanced HCC.

Biliary tract cancers (BTCs) include intrahepatic cholangiocarcinoma, extrahepatic cholangiocarcinoma, and gallbladder carcinoma [35]. Existing studies have shown that the prognosis of these types of cholangiocarcinomas and the effectiveness of treatments are different, especially for systematic treatment [36,37]. Cisplatin combined with gemcitabine is considered the standard first-line chemotherapy for advanced BTC [38]. However, in patients with ICC, the efficacy is unsatisfactory [39,40]. ICC, a type of primary liver cancer, can be treated in a similar manner as HCC [3]. Some studies have shown that TACE in locally advanced ICC can achieve curative effects similar to those of palliative surgical resection [41]. Postoperative adjuvant TACE, radiotherapy and other local treatments can significantly reduce tumour recurrence and improve survival [42,43]. For ICC patients with a hepatitis B virus infection background, postoperative antiviral therapy can significantly reduce tumour recurrence [44]. These findings are similar to those of many HCC treatments.

Compared with HCC, an effective treatment for ICC is even more lacking [2,3]. However, targeted PD-1 therapy has been approved for advanced ICC with microsatellite instability-high (MSI-H) or mismatch repair deficiency (dMMR). The efficacy of immune checkpoint therapy in mismatch repair proficient (pMMR) and microsatellite stable (MSS) ICC is still unclear, and the predictive effect of tumour mutation burden (TMB) and PD-L1 expression status is also inconclusive [45–47]. Furthermore, a new generation of bifunctional checkpoint inhibitors, M7824, can simultaneously target PD-L1 and TGF-β and further improve the effectiveness of immunotherapy by effectively inhibiting immune escape. A phase III clinical trial of M7824 combined with chemotherapy as a first-line treatment for advanced cholangiocarcinoma is in progress [48]. In the future, systemic treatment of ICC, including immunotherapy, can learn from the experience of HCC therapies. In the next few years, multicenter randomized-controlled studies on ICC immunotherapy will be an important trend in clinical research of advanced ICC. The combination of ICIs with chemotherapy, targeted therapy, and local treatment will be also a hot spot worthy of attention.

We admit that there are some shortcomings in this research. First, our study is a retrospective analysis, which has inevitable bias. Second, the sample size of this study was small, and the conclusions of the research were not strong enough. For example, the efficacy of several PD-1 inhibitors cannot be compared. Similarly, the best treatment cannot be distinguished among various combination treatments. Third, the ICC patients in our cancer centre had not undergone genetic testing. Since it is a trend to combine molecular markers to predict efficacy, molecular markers may better indicate the efficacy of immune or targeted therapy and suggest more suitable combination therapies. For example, the bifunctional checkpoint inhibitor M7824 can significantly benefit cholangiocarcinoma patients with positive expression of PD-L1 and TGF-β. Therefore, anti-PD-1 agents combined with molecular targeted inhibitors may achieve better efficacy in PD-1 positive patients.

In conclusion, PD-1-targeted immunotherapy is a safe and effective treatment for advanced ICC patients and provides another therapeutic strategy for these patients.

## Data Availability

The data that support the findings of this study are available from the corresponding author.

## References

[1] Sung H, Ferlay J, Siegel RL, et al. Global cancer statistics 2020: GLOBOCAN estimates of incidence and mortality worldwide for 36 cancers in 185 countries. CA Cancer J Clin. 2021;71(3):209–249. 10.3322/caac.21660 33538338

[2] Valle JW, Kelley RK, Nervi B, et al. Biliary tract cancer. Lancet. 2021;397(10272):428–444. 33516341 10.1016/S0140-6736(21)00153-7

[3] Sirica AE, Gores GJ, Groopman JD, et al. Intrahepatic cholangiocarcinoma: continuing challenges and translational advances. Hepatology. 2019;69(4):1803–1815. 30251463 10.1002/hep.30289 PMC6433548

[4] Patel T. Cholangiocarcinoma-controversies and challenges. Nat Rev Gastroenterol Hepatol. 2011;8(4):189–200. 21460876 10.1038/nrgastro.2011.20 PMC3888819

[5] Mavros MN, Economopoulos KP, Alexiou VG, et al. Treatment and prognosis for patients with intrahepatic cholangiocarcinoma: systematic review and meta-analysis. JAMA Surg. 2014;149(6):565–574. 24718873 10.1001/jamasurg.2013.5137

[6] Le DT, Uram JN, Wang H, et al. PD-1 blockade in tumors with mismatch-repair deficiency. N Engl J Med. 2015;372(26):2509–2520. 26028255 10.1056/NEJMoa1500596 PMC4481136

[7] Kansy BA, Concha-Benavente F, Srivastava RM, et al. PD-1 status in CD8+ T cells associates with survival and anti-PD-1 therapeutic outcomes in head and neck cancer. Cancer Res. 2017;77(22):6353–6364. 28904066 10.1158/0008-5472.CAN-16-3167 PMC5690836

[8] Migden MR, Rischin D, Schmults CD, et al. PD-1 blockade with cemiplimab in advanced cutaneous squamous-cell carcinoma. N Engl J Med. 2018;379(4):341–351. 29863979 10.1056/NEJMoa1805131

[9] Sato Y, Kinoshita M, Takemura S, et al. The PD-1/PD-L1 axis may be aberrantly activated in occupational cholangiocarcinoma. Pathol Int. 2017;67(3):163–170. 28139862 10.1111/pin.12511

[10] Ye Y, Zhou L, Xie X, et al. Interaction of B7-H1 on intrahepatic cholangiocarcinoma cells with PD-1 on tumor-infiltrating T cells as a mechanism of immune evasion. J Surg Oncol. 2009;100(6):500–504. 19697355 10.1002/jso.21376

[11] Gani F, Nagarajan N, Kim Y, et al. Program death 1 immune checkpoint and tumor microenvironment: Implications for patients with intrahepatic cholangiocarcinoma. Ann Surg Oncol. 2016;23(8):2610–2617. 27012989 10.1245/s10434-016-5101-y

[12] Ricci AD, Rizzo A, Brandi G. Immunotherapy in biliary tract cancer: worthy of a second look. Cancer Control. 2020;27(3):1073274820948047. 32806956 10.1177/1073274820948047 PMC7791443

[13] Boilève A, Hilmi M, Smolenschi C, et al. Immunotherapy in advanced biliary tract cancers. Cancers (Basel). 2021;13(7):1569. 33805461 10.3390/cancers13071569 PMC8036747

[14] Rizzo A, Ricci AD, Brandi G. Recent advances of immunotherapy for biliary tract cancer. Expert Rev Gastroenterol Hepatol. 2021;15(5):527–536. 33215952 10.1080/17474124.2021.1853527

[15] Guo X, Shen W. Latest evidence on immunotherapy for cholangiocarcinoma. Oncol Lett. 2020;20(6):381. 33154779 10.3892/ol.2020.12244 PMC7608025

[16] Rizzo A, Ricci AD, Brandi G. Durvalumab: an investigational anti-PD-L1 antibody for the treatment of biliary tract cancer. Expert Opin Investig Drugs. 2021;30(4):343–350. 10.1080/13543784.2021.1897102 33645367

[17] Nagtegaal ID, WHO Classification of Tumours Editorial Board, Odze RD, Klimstra D, et al. The 2019 WHO classification of tumours of the digestive system. Histopathology. 2020;76(2):182–188. 31433515 10.1111/his.13975 PMC7003895

[18] Eisenhauer EA, Therasse P, Bogaerts J, et al. New response evaluation criteria in solid tumours: revised RECIST guideline (version 1.1). Eur J Cancer. 2009;45(2):228–247. 19097774 10.1016/j.ejca.2008.10.026

[19] Lencioni R, Llovet JM. Modified RECIST (mRECIST) assessment for hepatocellular carcinoma. Semin Liver Dis. 2010;30(1):52–60. 20175033 10.1055/s-0030-1247132 PMC12268942

[20] Johnson PJ, Berhane S, Kagebayashi C, et al. Assessment of liver function in patients with hepatocellular carcinoma: a new evidence-based approach-the ALBI grade. J Clin Oncol. 2015;33(6):550–558. 25512453 10.1200/JCO.2014.57.9151 PMC4322258

[21] Chan A, Leung H, Chong C, et al. Validating the ALBI grade: Its current and future use in HCC prognostication. J Hepatol. 2017;66(3):661–663. 27890792 10.1016/j.jhep.2016.10.037

[22] Pinato DJ, Sharma R, Allara E, et al. The ALBI grade provides objective hepatic reserve estimation across each BCLC stage of hepatocellular carcinoma. J Hepatol. 2017;66(2):338–346. 27677714 10.1016/j.jhep.2016.09.008

[23] Deng M, Ng S, Cheung ST, et al. Clinical application of Albumin-Bilirubin (ALBI) score: the current status. Surgeon. 2020;18(3):178–186. 31601470 10.1016/j.surge.2019.09.002

[24] Brindley PJ, Bachini M, Ilyas SI, et al. Cholangiocarcinoma. Nat Rev Dis Primers. 2021;7(1):65. 34504109 10.1038/s41572-021-00300-2 PMC9246479

[25] Lee SH, Lee HS, Lee SH, et al. Efficacy and safety of pembrolizumab for gemcitabine/Cisplatin-Refractory biliary tract cancer: a multicenter retrospective study. J Clin Med. 2020;9(6):1769. 10.3390/jcm9061769 PMC7355970 32517311

[26] Kang J, Jeong JH, Hwang HS, et al. Efficacy and safety of pembrolizumab in patients with refractory advanced biliary tract cancer: tumor proportion score as a potential biomarker for response. Cancer Res Treat. 2020;52(2):594–603. 32019287 10.4143/crt.2019.493 PMC7176957

[27] Pinato DJ, Kaneko T, Saeed A, et al. Immunotherapy in hepatocellular cancer patients with mild to severe liver dysfunction: adjunctive role of the ALBI grade. Cancers (Basel). 2020;12(7):1862. 10.3390/cancers12071862 PMC7408648 32664319

[28] Wong J, Kwok G, Tang V, et al. Ipilimumab and nivolumab/pembrolizumab in advanced hepatocellular carcinoma refractory to prior immune checkpoint inhibitors. J Immunother Cancer. 2021;9(2):e001945. 33563773 10.1136/jitc-2020-001945 PMC7875295

[29] Hilmi M, Neuzillet C, Calderaro J, et al. Angiogenesis and immune checkpoint inhibitors as therapies for hepatocellular carcinoma: current knowledge and future research directions. J Immunother Cancer. 2019;7(1):333. 31783782 10.1186/s40425-019-0824-5 PMC6884868

[30] D'Andrea MA, Reddy GK. Systemic immunostimulatory effects of radiation therapy improves the outcomes of patients with advanced NSCLC receiving immunotherapy. Am J Clin Oncol. 2020;43(3):218–228. 31842114 10.1097/COC.0000000000000651

[31] Bang YJ, Golan T, Dahan L, et al. Ramucirumab and durvalumab for previously treated, advanced non-small-cell lung cancer, gastric/gastro-oesophageal junction adenocarcinoma, or hepatocellular carcinoma: an open-label, phase Ia/b study (JVDJ). Eur J Cancer. 2020;137:272–284. 32827847 10.1016/j.ejca.2020.06.007

[32] Zhu AX, Finn RS, Edeline J, et al. Pembrolizumab in patients with advanced hepatocellular carcinoma previously treated with sorafenib (KEYNOTE-224): a non-randomised, open-label phase 2 trial. Lancet Oncol. 2018;19(7):940–952. 29875066 10.1016/S1470-2045(18)30351-6

[33] Finn RS, Qin S, Ikeda M, et al. Atezolizumab plus bevacizumab in unresectable hepatocellular carcinoma. N Engl J Med. 2020;382(20):1894–1905. 32402160 10.1056/NEJMoa1915745

[34] Lai E, Astara G, Ziranu P, et al. Introducing immunotherapy for advanced hepatocellular carcinoma patients: too early or too fast. Crit Rev Oncol Hematol. 2021;157:103167. 33271389 10.1016/j.critrevonc.2020.103167

[35] Razumilava N, Gores GJ. Cholangiocarcinoma. Lancet. 2014;383(9935):2168–2179. 24581682 10.1016/S0140-6736(13)61903-0 PMC4069226

[36] Banales JM, Marin J, Lamarca A, et al. Cholangiocarcinoma 2020: the next horizon in mechanisms and management. Nat Rev Gastroenterol Hepatol. 2020;17(9):557–588. 32606456 10.1038/s41575-020-0310-z PMC7447603

[37] Massironi S, Pilla L, Elvevi A, et al. New and emerging systemic therapeutic options for advanced cholangiocarcinoma. Cells. 2020;9(3):688. 10.3390/cells9030688 PMC7140695 32168869

[38] Valle J, Wasan H, Palmer DH, et al. Cisplatin plus gemcitabine versus gemcitabine for biliary tract cancer. N Engl J Med. 2010;362(14):1273–1281. 20375404 10.1056/NEJMoa0908721

[39] Ebata T, Bile Duct Cancer Adjuvant Trial (BCAT) Study Group, Hirano S, Konishi M, et al. Randomized clinical trial of adjuvant gemcitabine chemotherapy versus observation in resected bile duct cancer. Br J Surg. 2018;105(3):192–202. 29405274 10.1002/bjs.10776

[40] Edeline J, Benabdelghani M, Bertaut A, et al. Gemcitabine and oxaliplatin chemotherapy or surveillance in resected biliary tract cancer (PRODIGE 12-ACCORD 18-UNICANCER GI): a randomized phase III study. J Clin Oncol. 2019;37(8):658–667. 30707660 10.1200/JCO.18.00050

[41] Scheuermann U, Kaths JM, Heise M, et al. Comparison of resection and transarterial chemoembolisation in the treatment of advanced intrahepatic cholangiocarcinoma–a single-center experience. Eur J Surg Oncol. 2013;39(6):593–600. 23611755 10.1016/j.ejso.2013.03.010

[42] Li J, Wang Q, Lei Z, et al. Adjuvant transarterial chemoembolization following liver resection for intrahepatic cholangiocarcinoma based on survival risk stratification. Oncologist. 2015;20(6):640–647. 25956404 10.1634/theoncologist.2014-0470 PMC4571785

[43] Hammad AY, Berger NG, Eastwood D, et al. Is radiotherapy warranted following intrahepatic cholangiocarcinoma resection? The impact of surgical margins and lymph node status on survival. Ann Surg Oncol. 2016;23(S5):912–920. 27654107 10.1245/s10434-016-5560-1

[44] Sha M, Jeong S, Xia Q. Antiviral therapy improves survival in patients with HBV infection and intrahepatic cholangiocarcinoma undergoing liver resection: novel concerns. J Hepatol. 2018;68(6):1315–1316. 29475065 10.1016/j.jhep.2018.01.039

[45] Ueno M, Ikeda M, Morizane C, et al. Nivolumab alone or in combination with cisplatin plus gemcitabine in japanese patients with unresectable or recurrent biliary tract cancer: a non-randomised, multicentre, open-label, phase 1 study. Lancet Gastroenterol Hepatol. 2019;4(8):611–621. 31109808 10.1016/S2468-1253(19)30086-X

[46] Piha-Paul SA, Oh DY, Ueno M, et al. Efficacy and safety of pembrolizumab for the treatment of advanced biliary cancer: results from the KEYNOTE-158 and KEYNOTE-028 studies. Int J Cancer. 2020;147(8):2190–2198. 32359091 10.1002/ijc.33013

[47] Kim RD, Chung V, Alese OB, et al. A phase 2 multi-institutional study of nivolumab for patients with advanced refractory biliary tract cancer. JAMA Oncol. 2020;6(6):888–894. 32352498 10.1001/jamaoncol.2020.0930 PMC7193528

[48] Yoo C, Oh DY, Choi HJ, et al. Phase I study of bintrafusp alfa, a bifunctional fusion protein targeting TGF-β and PD-L1, in patients with pretreated biliary tract cancer. J Immunother Cancer. 2020;8(1):e000564. 32461347 10.1136/jitc-2020-000564 PMC7254161

